# Evaluating the benefits of neoadjuvant chemotherapy for advanced epithelial ovarian cancer: a retrospective study

**DOI:** 10.1186/s13048-019-0562-9

**Published:** 2019-09-13

**Authors:** Yan Gao, Yuan Li, Chunyu Zhang, Jinsong Han, Huamao Liang, Kun Zhang, Hongyan Guo

**Affiliations:** Department of Obstetrics & Gynecology, Peking University Third Hospital, No. 49 Huayuan North Road, Haidian District, Beijing, 100191 China

**Keywords:** Advanced ovarian epithelial Cancer, Neoadjuvant chemotherapy, Primary Debulking surgery, Chemoresistance, Prognosis

## Abstract

**Objective:**

To compare the chemoresistance and survival in patients with stage IIIC or IV epithelial ovarian cancer who were treated with neoadjuvant chemotherapy (NACT) followed by interval debulking surgery (IDS) or primary debulking surgery (PDS). The clinical characteristics of patients who benefited from NACT were further evaluated.

**Methods:**

We retrospectively analyzed 220 patients who underwent NACT followed by IDS or PDS from January 2002 to December 2016. Differences in clinicopathological features, chemoresistance and prognosis were analyzed.

**Results:**

The incidence rate for optimal cytoreduction and chemoresistance in the NACT group was relatively higher than PDS group. No differences were observed in progression free survival or overall survival. Patients without macroscopic RD in NACT group (NACT-R0) had a similar prognosis compared to those in PDS group who had RD<1 cm, and a relatively better prognosis compared to the PDS group that had RD ≥ 1 cm. The survival curve showed that patients in NACT-R0 group that were chemosensitive seemed to have a better prognosis compared to patients in PDS group that had RD.

**Conclusion:**

Patients without RD after PDS had the best prognosis, whereas patients with RD after NACT followed by IDS had the worst. However, even if patients achieved no RD, their prognosis varied depending on chemosensitivity. Survival was better in patients who were chemosensitive compared to thosewho underwent PDS but had RD. Hence evaluating the chemosensitivity and feasibility of complete cytoreduction in advance is crucial.

## Introduction

Epithelial ovarian cancer (EOC) is the most lethal gynecologic malignancy and is closely associated with tumor recurrence and chemoresistance [1]. Approximately 75% of patients are diagnosed at an advanced stage with widespread peritoneal lesions and frequent chemoresistance [2]. Retrospective studies have demonstrated a strong association between the size of post-surgical residual tumor and prognosis [3]. At present, the gold standard for management is primary debulking surgery followed by platinum-based adjuvant chemotherapy. However, optimal debulking can be achieved in only 30–60% of stage III/IV ovarian cancers [4–7]. Neoadjuvant chemotherapy followed by interval debulking surgery has been proposed as an alternative to conventional PDS. These indications are for patients with a low probability of optimal cytoreduction or reversible contraindications [8].

However, the role of NACT and PDS is still controversial. Several studies, including randomized controlled trials, have reported a significantly higher optimal debulking rate in patients who underwent NACT-IDS but a similar overall survival between NACT and PDS [9–13]. Several retrospective studies regarding stage IV ovarian cancer patients have reported that the survival outcomes in patients who underwent PDS were lower compared to patients who underwent NACT [14, 15]. In addition, several meta-analyses have suggested that NACT is associated with worst outcomes when compared to standard PDS followed by adjuvant chemotherapy [16–19].

Additionally, emerging evidence from recent studies showed a higher incidence of platinum-resistance after NACT at first relapse compared to patients who received PDS [20–22]. However, studies by Rauh-Hain [23] and da Costa [24] reported that the incidence of platinum-resistance after NACT at first relapse was similar, whereas the risk of platinum-resistance at second relapse was higher: Rauh-Hain’s (adjusted OR 4.06, *P* = 0.001) and da Costa’s (adjusted HR 1.92, *P* = 0.009).

This has led us to wonder if the controversial results were relevant to individual differences. This hypothesis may explain why a small subset of patients failed to respond well during NACT and could not achieve optimal debulking by IDS, and thus poor prognosis. Even for the majority of patients who attained optimal debulking after NACT, due to differences in chemosensitivity, patient outcomes varied. In addition, patients who demonstrated initial chemosensitivity with complete debulking would still relapse. Hence, we intend to retrospectively compare the chemoresistance and survival between NACT-IDS and PDS. And The clinical characteristics of patients who benefited from NACT were further evaluated.

## Methods

### Patient population and selection criteria

We conducted a retrospective review of all patients diagnosed with EOC and who received debulking surgery in Peking University Third Hospital from January 2002 to December 2016. Written informed consents were obtained from all patients. Institutional Ethics Committee of Peking University approved this study in March 2018([2018]107–01).

Patients with International Federation of Gynecology and Obstetrics (FIGO) stages IIIC and IV EOC by laparoscopy/ laparotomy and who underwent platinum based chemotherapy were included in this study. A total of 220 patients were selected, of which, 79 underwent NACT followed by IDS and 141 underwent PDS followed by adjuvant chemotherapy. Prior to NACT, 36 of the 79 patients were diagnosed histologically by laparoscopy, and the remaining 43 patients were diagnosed using cytology samples obtained from the abdominal or thoracic paracentesis. During IDS, histology confirmation was performed for all patients. NACT patients were administered 3–6 cycles of platinum based chemotherapy followed by interval debulking surgery, and then again with at least three additional cycles of platinum-based chemotherapy. For patients who underwent PDS, at least six cycles of platinum-based chemotherapy was administered.

### Data collection and follow up

Clinical information regarding pre-surgical characteristics including age, stage, histology type, histology grade, pleural effusion, comorbidities, serum CA-125, and computerized tomography (CT) images were obtained from patients’ records. Surgical findings were documented using a standardized form that specified the sites and size of the initial and residual tumor, as well as the surgical procedures that were performed. Optimal cytoreduction was defined as residual tumor no larger than 1 cm. R0 was defined as no residual tumor. R1 was defined as the size of the RD<1 cm. R2 was defined as the size of the RD ≥ 1 cm. A localized tumor spread pattern was defined according to the following: with or without multifocal lesions at either one of the following sites (including the peritoneum, mesenterium, diaphragm, recto-uterine fossa, and intestines); or unifocal lesions with less than 5 sites. A diffuse tumor spread pattern was defined as follows: omental cake, multiple nodules at more than two different sites (including the peritoneum, mesenterium, diaphragm, recto-uterine fossa, and intestines); or unifocal lesions at more than 5 sites [25].

During follow-up, recurrence or progression was defined by evidence of recurrence or progressive disease through imaging or pathology confirmation on biopsy, or based on serum CA125 levels according to RECIST1.1 and agreed to the Gynecological Cancer Intergroup (GCIG) definition as greater than, or equal to, two times the upper limit of the reference range on two occasions at least 1 week apart [26]. Chemo-resistance was defined as recurrence after achieving complete recovery after initial treatment in less than 6 months, or progression of the disease during chemotherapy. Chemosensitive patients were defined as have recurrence after achieving complete recovery after initial treatment after 6 months. Overall survival (OS) was calculated from date of treatment (surgery or chemotherapy) to date of death or date of last follow-up (end of follow-up 8th February 2018). Progression-free survival (PFS) was defined as the time from treatment (surgery or chemotherapy) to physical, biological or radiological evidence of disease progression, or death as a result from any cause. Thirteen patients were lost during follow-up. The median follow-up period was 67.8 months.

### Statistical analysis

The baseline characteristics were compared using Student’s t test or Chi-square test. Clinical factors were evaluated for their association with chemosensitivity using logistic regression models. Overall survival (OS) and progression-free survival (PFS) were analyzed using the Kaplan–Meier method. The log-rank test was used to investigate the difference in survival between the two groups. Cox proportional hazard analysis was used to evaluate the prognostic factors that affected survival. Statistical analyses were performed using SPSS software version 19.0 (SPSS Inc., Chicago, IL, USA). *P* values<0.05 were considered statistically significant.

## Results

### Baseline characteristics of patients in the NACT and PDS group

Among the 220 patients, 141 underwent PDS and post-surgical chemotherapy and 79 underwent NACT followed by interval debulking surgery. The majority of the patients in the NACT group were in the FIGO stage IV (24.1 vs.15.6%), but no statistical significant differences were found between the two groups (*P* = 0.123). The incidence rates for pretreatment CA125 levels over 500 U/ml, pleural metastasis, and liver metastasis were significantly higher in the NACT group compared to the PDS group. The baseline characteristics of the patients in both groups are shown in Table 1.

**Table 1:** Comparision of the clinicopathological characteristics between the 220 patients in the NACT-IDS and PDS group with stage IIIC and IV epithelial ovarian cancer

Characteristics	PDS group (*n* = 141)	NACT group(*n* = 79)	*P* value
Age (years), mean ± SD	55.99 ± 11.10	57.08 ± 10.38	0.526
Pathology type			0.390
Serous carcinoma	117(83.0%)	69(87.3%)	
Other types	24(17.0%)	10(12.7%)	
Stage			0.123
IIIC	119(84.4%)	60(75.9%)	
IV	22(15.6%)	19(24.1%)	
Histology grade*			0.035
G1	2(1.4%)	0(0%)	
G2	27(19.4%)	6(7.7%)	
G3	110(79.1%)	72(92.3%)	
Initial CA125 level*			0.036
<500 U/ml	60(42.6%)	22(28.2%)	
≥ 500 U/ml	81(57.4%)	56(71.8%)	
Pleural effusion	12(8.5%)	14(17.7%)	0.042
Liver metastasis	15(10.6%)	6(7.6%)	0.461
Tumor distribution			< 0.001
Localized	38(27.0%)	0 (0%)	
Diffuse	103(73.0%)	79 (100%)	
lymphadenectomy	107(75.9%)	60(75.9%)	0.992
Residual disease			0.071
None, R0	46 (32.6%)	34(43.0%)	
<1 cm,R1	44 (31.2%)	28(35.4%)	
≥ 1 cm,R2	51 (36.2%)	17(21.5%)	
Lymph node metastasis*	69(61.1%)	20(32.3%)	< 0.001

Histology grade* refers to 3 cases with unknown histology grade due to difficult histology recognition

Initial CA125 level* refers to 1 case with unknown Initial CA125 level

Lymph node metastasis* refers to 28 cases in PDS group and 17 cases in NACT group respectively had unknown lymph node accessment as they didn't undergo lymphadenectomy

Compared to the sites and size of the initial tumor, patients in the NACT group had diffused tumor dissemination, while patients in the PDS group had relatively localized tumors. Multiple mesenteric nodules, omental cake, and multiple liver metastases were observed in the NACT group and showed significant differences to the PDS group (Table 2).

**Table 2:** Parameters for tumor spread between the PDS and NACT group

Parameter	PDS group	NACT group	*P* value
Pelvic mass ≥ 10 cm	18.4% (26/141)	16.5% (13/79)	0.712
Large volume Ascites (>500 mL)	45.4% (64/141)	45.6% (36/79)	0.980
Multiple peritoneal nodules	56.7%(80/141)	57.0% (45/79)	0.974
Multiple mesenteric nodules	27.0%(38/141)	48.1% (38/79)	0.002
Multiple diaphragm nodules	30.5%(43/141)	43.0% (11/56)	0.061
Omental cake	22.0%(31/141)	79.7% (63/79)	< 0.001
Multisectional intestinal infiltration ≥3 segments	23.4%(33/141)	12.7% (10/79)	0.054
Multiple liver metastasis	14.9%(21/139)	27.8% (22/79)	0.020
Fusion of enlarged lymph node	4.4%(5/113)	1.6% (1/62)	0.226

### Optimal debulking rate and residual diseases comparison between the NACT and PDS group

The incidence rate for optimal cytoreduction (R0 + R1) in the NACT group was relatively higher compared to patients in the PDS group (78.5% vs 63.8%, *P* = 0.024). The rate of “no residual disease” (R0) was relatively higher in the NACT group compared to the PDS group, but was not significantly different (43.0 vs. 32.6%; *P* = 0.123) (Additional file 1: Table S1).

Residual disease (RD) less than 1 cm (R1) in the NACT and PDS groups were mostly localized in the diaphragm (46.4% vs 40.9%), intestine (39.3% vs 38.6%), and peritoneum (39.3% vs 36.4%), and were characterized by massive military nodules. RD more than 1 cm (R2) were characterized as follows: multisectional intestinal invasion (≥3 segments) (23.5% vs 12.7%); peritoneal diffuse dissemination of tumor nodules up to the diaphragm (29.4% vs 33.3%), contracted omentum with dense adhesion to surrounding organs (23.5% vs 9.8%), or a dense adhesion and fusion of enlarged lymph nodes (4.4% vs 1.6%). However, sites for RD showed no differences between the two groups (*P* > 0.05) .

### Chemoresistance rates between the NACT and PDS groups

During follow-up, 29.5% (61/207) of patients experienced platinum-resistant recurrence, 40.3% (31/77) of which were in the NACT group and 23.1% (30/130) were in the PDS group (*P* = 0.009). As shown in Additional file 2: Table S2, univariate analyses found that histology type (*P* = 0.009), massive ascites (*P* = 0.011), diffuse disseminated disease (*P* = 0.001), macroscopic RD (*P* = 0.006), and NACT (*P* = 0.009) were factors that increased the risk of platinum-resistance. Variables with *p* values < 0.05 in the univariate analyses were included in the final multivariate logistic regression analyses. NACT (*P* = 0.012) and non-serous carcinoma (*P* = 0.018) were independent risk factors for chemoresistance (Additional file 3: Table 3).

### Analysis of patient survival between the NACT and PDS groups

#### No difference was observed in PFS or OS between the NACT and PDS groups

Median follow-up period was 67.8 months (range 34.2–147.5 months). Patients were analyzed based on treatment groups. The median PFS was 19.9 months (95% CI 13.4–26.4) for the NACT group and 25.4 months (95% CI 21.0–29.7) for the PDS group. No statistically significant differences were found between the two groups (*p* = 0.371). The median OS was 52.3 months (95% CI 39.9–64.7) for the NACT group, and 67.8 months (95% CI 53.0–82.6) for the PDS group (*p* = 0.209) See Additional file 4: Figure S1.

#### Patients in the NACT group with residual disease experienced worse PFS and OS compared to patients in the PDS group

We compared the PFS and OS based on treatment selection (NACT or PDS) and the size of the residual disease (RD). The median PFS for women with no RD (R0), with RD less than 1 cm in diameter (R1), and with RD larger than 1 cm (R2) between the NACT and PDS groups were 23.5 vs 50.2, 19.2 vs 25.4, and 13.8 vs 19.1 months, respectively (*p* > 0.05). The median OS (NACT group vs PDS group) for women with R0, R1, R2 was 68.2 vs 106.2, 50.0 vs 61.3, and 42.3 vs 41.8 months, respectively (*p* > 0.05)(Additional file 5: Table 4).

In the NACT group, the PFS and OS were relatively longer in the subgroups with no RD compared to patients with RD, however no significant differences were found (*P* > 0.05). While, in the PDS group, the PFS and OS were significantly longer in the subgroups with R0 compared to patients with R1 (*P* = 0.011), however, no significant differences between the subgroups with R1 and R2 (*P* > 0.05) were found (Additional file 6: Figure S2).

#### Patients who were chemosensitive and without residual disease seemed to benefit from NACT

**Fig. 1:**
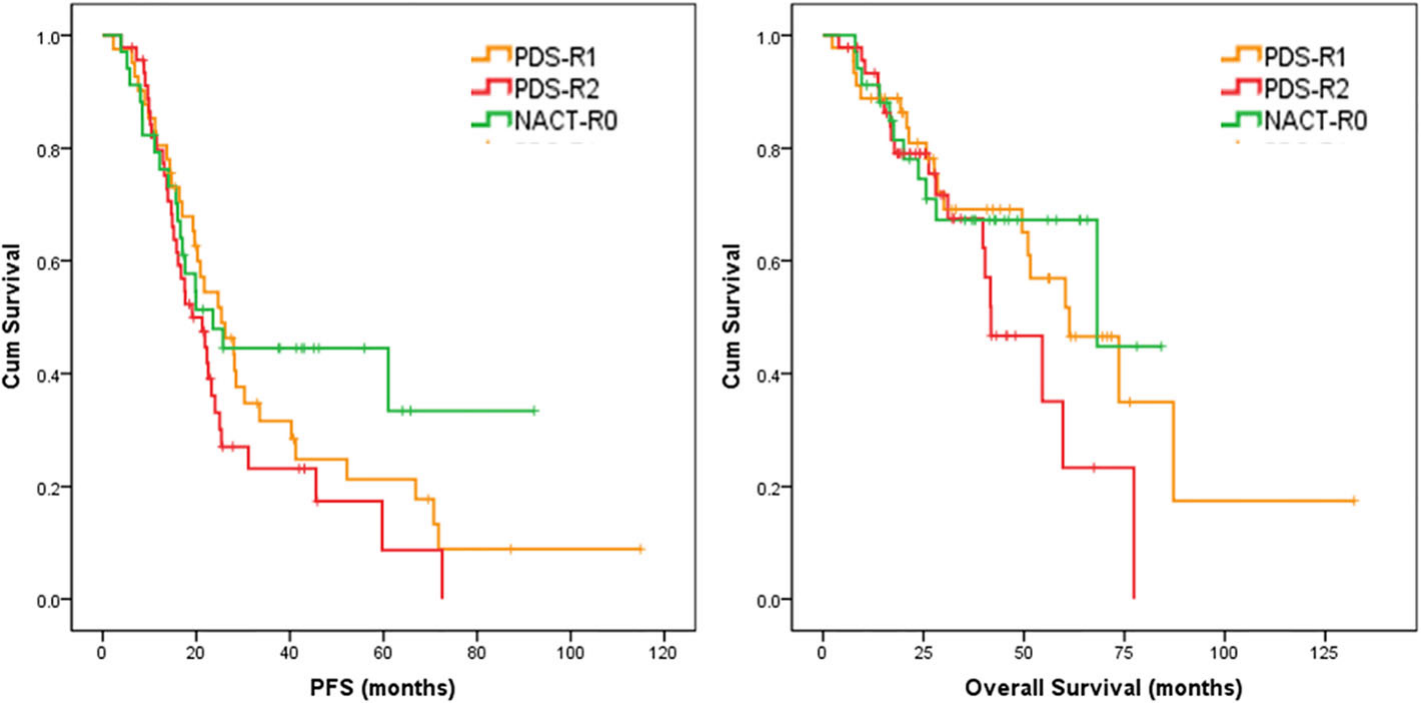
Kaplan–Meier survival curve for PFS(left) and OS(right) for patients with no residual tumor in the NACT group (NACT-R0), patients with residual tumor less than 1 cm in the PDS-R1 subgroup and with residual tumor larger than 1 cm in the PDS-R2 subgroup

**Fig. 2:**
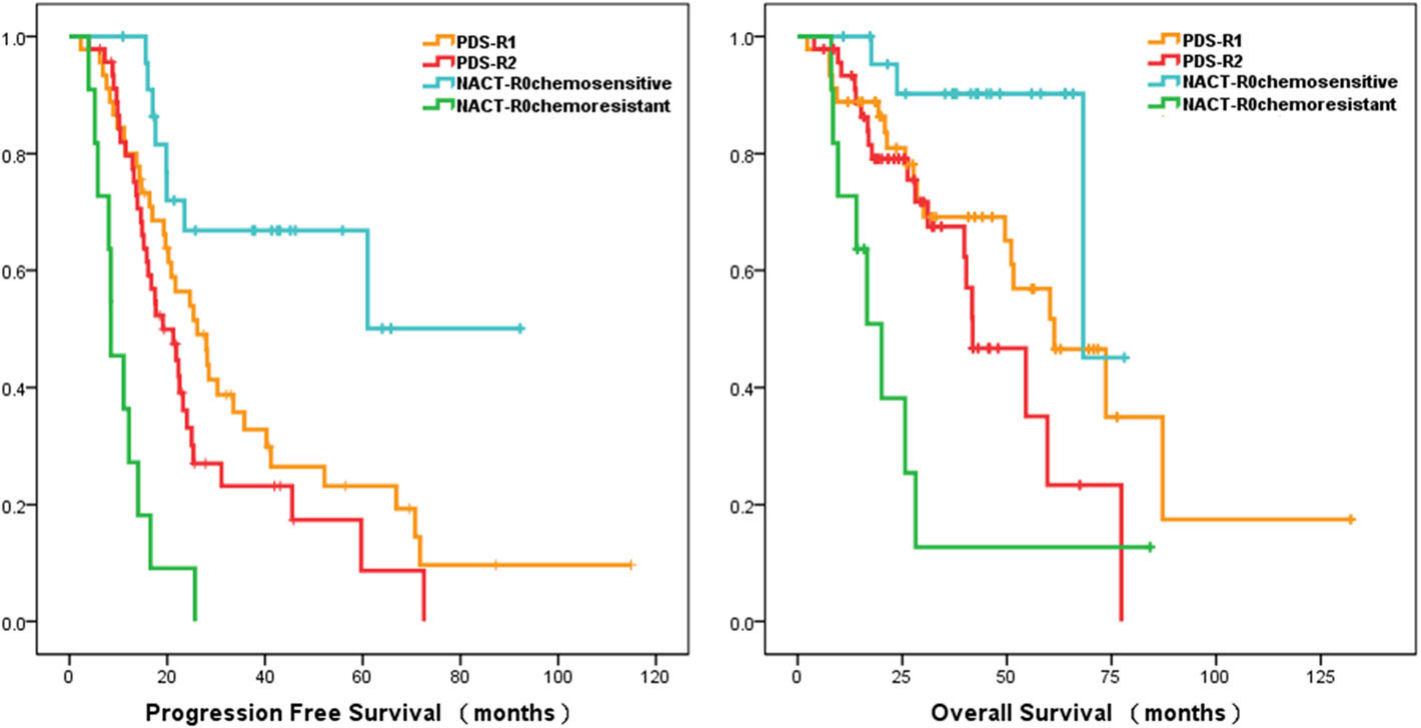
Kaplan–Meier survival curve for PFS(left) and OS(right) for patients with no residual tumor in the NACT group (NACT-R0) based on chemosensitivity, patients with residual tumor less than 1 cm in the PDS-R1 subgroup and with residual tumor larger than 1 cm in the PDS-R2 subgroup

Although there were no statistical differences, patients with no RD in the NACT group (NACT-R0) had a similar prognosis compared to patients in the PDS group that had RD < 1 cm (PDS-R1) (PFS: 23.5 vs. 25.4 months, *p* = 0.431; OS: 68.2 vs. 61.3 months, *p* = 0.728), and a relatively better prognosis compared to patients in the PDS group that had RD ≥ 1 cm (PDS-R2) (PFS: 23.5 vs. 19.1 months, *p* = 0.097; OS: 68.2 vs. 41.8 months, *p* = 0.172)(Fig. 1). Patients in the NACT-R0 subgroup that were chemoresistant had evidently a worse prognosis compared to patients in the PDS-R2 group, and the results were statistically significant (PFS: 8.5 vs. 19.1 months, *p* < 0.001; OS: 20.1 vs. 41.8 months, *p* = 0.025). However, the survival curve demonstrated that patients in NACT-R0 group that were chemosensitive seemed to have a better prognosis compared to patients in the PDS group that had RD (both PDS-R1 and PDS-R2) (Fig. 2).

As shown in Additional file 7: Table S5 and Additional file 8: Table S6, univariate analyses found that chemoresistance (*P* = 0.000), and macroscopic RD (*P* = 0.004) were risk factors for PFS; whereas massive ascites (*P* = 0.008), macroscopic RD (*P* = 0.003), and chemoresistance (*P* = 0.000) were risk factors for OS. Using backward elimination Multivariable Cox regression model (from Additional file 9: Table S7 and Additional file 10: Table S8) identified chemoresistance (*P* < 0.001) and macroscopic RD (*P* = 0.030) as independent predictors for survival.

## Discussion

Optimal cytoreduction is a critical prognostic factor for prolonged survival, whether it is performed before or after chemotherapy. Recent studies have suggested that NACT have reduced morbidity after surgery and are more suitable for optimal debulking surgery [9, 11–13, 27, 28]. NACT followed by IDS (NACT-IDS) is considered to be an alternative to conventional PDS for treating advanced ovarian cancer patients and expected to have better prognosis. With the increased use of NACT, debates have risen as to whether NACT-IDS offers any benefits.

In line with several studies, we observed a dramatic increase in optimal cytoreductive rates in patients treated with NACT-IDS compared to PDS. However no survival benefits were observed. The majority of studies have demonstrated similar progression free survival (PFS) and overall survival (OS) between patients treated with NACT and PDS [9–13]. In our study, the patients’ baseline characteristics were well balanced. We found a higher rate of optimal cytoreduction for patients in the NACT group, but showed no improvement in PFS and OS compared to patients in the PDS group (median PFS: 19.9 months vs 25.4 months, *P* = 0.371; median OS: 52.3 months vs 67.8 months, *P* = 0.209). This observation is consistent with the results observed in the majority of previous studies.

An explanation for this is that chemoresistance in ovarian cancer may be induced by NACT, thus leading to a worse prognosis if recurrence occurs. Three retrospective trials demonstrated that patients receiving NACT-IDS had a higher risk of platinum-resistant recurrence compared to patients who underwent PDS [20–22]. However, it still remains unclear whether NACT could induce platinum-resistance. Our study showed that patients in the NACT-IDS group had a higher incidence of platinum-resistance at first relapse compared to patients in the PDS group, and was confirmed using multivariate regression analysis (adjusted OR 2.837, *P* = 0.002).

To explain this finding, one possible hypothesis is that the larger the volume of cancer present when chemotherapy is initiated, the higher the likelihood of development of mutations and chemoresistance [29]. Large bulky tumors are often necrotic and hypoxic. Their poor blood supply does not often lend itself to maximal intravenous or intraperitoneal chemotherapy delivery. Comparatively, smaller tumors are well perfused and easier to target. At the time of initial treatment, both chemosensitive and chemoresistant cells are present in the patient. Primary surgery decreases the tumor burden for both types of cells and decreases the amount of cells that could spontaneously mutate to chemoresistant phenotypes, and thus to some extent, reduce chemoresistance. However, by initiating chemical debulking first using the NACT approach, tumor cells have more time to build resistance. Introducing IDS in the middle of the six to eight cycles of chemotherapy may have an effect in decreasing tumor burden. However, patients are more susceptible to develop mutations and acquire chemoresistance due to the exposure of a larger tumor burden to chemotherapy before IDS [29].

In addition, our study demonstrated that residual disease was another risk factor that was associated with higher platinum-resistant recurrence risk (adjusted OR 2.575, *P* = 0.013). This is consistent with Rauh-Hain [23] and Lim [27]’s study suggesting that NACT enriches for cancer stem cells that exists in residual disease, which eventually leads to chemoresistance. Although the diameter of metastatic tumors could be reduced using NACT, even to less than 1 cm, the residual tumor tissue may contain cancer stem cells responsible for chemoresistant recurrence [20, 27, 30]. It has been reported that a small subset (5 to 12%) of patients show pathologic complete response (pCR) with no residual tumor cells after NACT and excellent prognosis [31, 32]. Hence, the crucial goal for NACT is to achieve no residual tumor after IDS or even pCR. Research should focus on finding therapies that increase pCR in patients.

Additionally, our data demonstrated that PDS patients debulked to no RD had the longest PFS and OS. For PDS patients debulked to RD<1 cm or to RD ≥ 1 cm, their prognosis had no significant differences. Although patients who were debulked to no RD after NACT (NACT-R0) had a significantly lower PFS and OS compared to patients with no RD by PDS (PDS-R0), they were comparable to patients with RD < 1 cm at PDS (PDS-R1). In addition these patients showed a relatively better outcome compared to patients with RD ≥ 1 cm at PDS (PDS-R2). However for patients who were debulked to RD < 1 cm (NACT-R1) or ≥ 1 cm (NACT-R2) after NACT, their prognosis were no better than patients with RD ≥ 1 cm at PDS (PDS-R2) .

These results suggest that, R0 resection at PDS was associated with the best prognosis. Debulking to RD < 1 cm provides a smaller but still significant benefit for patients with PDS. For these patients, aggressive surgical procedures should be performed if the tumor could be safely resected to microscopic levels. For patients following NACT who ended up with RD after IDS, their prognosis was usually quite poor. They were not able to achieve optimal cytoreduction at PDS. If NACT could have some effect on minimizing tumors to a certain level, resectability should be considered. However, due to the large tumor burden, the majority of the patients developed chemoresistance and showed no improvement after NACT. If chemoresistance could be predicted in advanced, PDS may be a better choice. Effective alternatives such as second-line chemotherapy, immune-therapy, targeted therapy or relevant clinical trials should also be recommended for these patients.

Patients debulked to no RD after NACT failed to show any evident benefits on survival compared to patients with macroscopic RD at PDS. We further compared the survival curves for patients in the NACT group without RD using subgroups based on chemosensitivity. Our results suggested that, among patients with no RD after NACT, survival was significantly better if the patients were chemosensitive compared to patients with RD at PDS.

Hence it is critical to identify these patients who may benefit from NACT for the assessment of complete resectability and chemosensitivity. In terms of unresectability, our study showed that NACT may not achieve benefits. These included multisectional intestinal invasion or severe pelvic adhesion with diffuse dissemination of tumor nodules, or a contracted omentum with dense adhesion to surrounding organs. Several methods have been proposed in the literature for pre-surgical evaluation, including CA125 levels, radiologic methods, peritoneal cancer index (PCI), and laparoscopic scoring [33–37]. The ASCO and SGO practice guidelines regarding the use of NACT do advocate laparoscopy as a tool to predict surgical resectability^.^ Relevant studies have suggested several laparoscopic and CT scoring systems that had certain predictive values for optimal cytoreduction. However limitations of over-or-underestimation were present in these methods [33–37]. With the improvement of surgical techniques, several previous ‘unresectable’ tumors could now be removed meticulously. Amendments and validation for a revised scoring system is now needed. More reliable predictors for resectability are essential for classifying tumors preoperatively and should be the subject of further study. In the 2018 SGO annual meeting on women’s cancer, Dr. Beryl suggested a new prospective. If R0 was not attained, low volume disease confined to single anatomic locations (≤1 cm-SL) may be an alternative [38]. The study showed that patients with RD ≤ 1 cm involving multiple anatomic locations (≤1 cm-ML) had similar outcomes to suboptimal debulked (RD>1 cm) patients [38]. Moving beyond complete cytoreduction, low volume RD may be another option for consideration.

Apart from resectability, platinum resistance should be taken into consideration before the use of NACT for AOC patients. Recognition of chemoresistant properties based on initial tumor biology and molecular phenotype in advance is complicated. Studies have demonstrated that tumor burden, tumor biology and even adjuvant chemotherapy itself was associated with chemoresistance. Gene mutations, include TP53, BRCA1/2, BRAF, KRAS, β-catenin, PTEN et al. [39, 40] have been considered to be associated with ovarian carcinogenesis and chemoresistance. In addition, the predictive value of cancer stem cell related markers, like CD44, ALDH1 have been demonstrated for chemoresistance [30]. However, there is still no single gene marker that have showed specificity, sensitivity, accuracy and effectiveness for chemosensitive assessment. A gene panel consisting of multiple genes may have the potential for chemoresistant evaluation. Further studies concerning the possible mechanism for acquired platinum resistance are needed.

There were several limitations to our study. First, this was a retrospective study that inevitably had a selection bias, as a small number of patients were excluded due to incomplete clinical data or lost during follow-up. In addition, patient selection for NACT or PDS relied on gynecological estimation and imaging. Secondly, due to limited patient numbers and the relatively short period of follow-up, death or progression only happened in a few patients. A larger cohort prospective study is currently in progress to validate our findings.

## Conclusion

Our study demonstrated no significant differences in PFS or OS between patients in the NACT and PDS groups. Patients without RD after PDS had the best prognosis, whereas patients with RD after NACT followed by IDS had the worst outcome. However, even if no RD was achieved at IDS, the use of NACT may benefit a selected group of patients, as their prognosis varied depending on chemosensitivity. For chemoresistant patients who underwent NACT, their prognosis were even worse compared to patients with RD > 1 cm after PDS. Our findings suggest that among women with no RD at IDS following NACT, survival was better for patients who were chemosensitive compared to patients who underwent PDS but with macroscopic RD. Thus, evaluating chemosensitivity and the feasibility of complete cytoreduction in advance is essential.

## Supplementary information

**Additional file 1: Table S1.** Size comparison of the residual tumor between the PDS and NACT group. (DOCX 15 kb)

**Additional file 2: Table S2.** Univariate analysis of risk factors for platinum resistance recurrence after NACT-IDS and PDS. (DOCX 18 kb)

**Additional file 3: Table S3.** Multivariate analysis of risk factors for platinum resistance recurrence after NACT-IDS and PDS. (DOCX 16 kb)

**Additional file 4: Figure S1.** Kaplan–Meier survival curves for PFS(left) and OS(right) for the NACT and PDS groups. (TIF 285 kb)

**Additional file 5: Table S4.** Prognosis comparison between the subgroups based on residual tumor for the PDS and NACT groups. (DOCX 16 kb)

**Additional file 6: Figure S2.** Kaplan–Meier survival curve for PFS(left) and OS(right) between the subgroups based on treatment selection and residual tumor. (TIF 478 kb)

**Additional file 7: Table S5.** Univariate analysis of risk factors for PFS after NACT-IDS and PDS. (DOCX 18 kb)

**Additional file 8: Table S6.** Univariate analysis of risk factors for OS after NACT-IDS and PDS. (DOCX 19 kb)

**Additional file 9: Table S7.** Multivariate analysis of risk factors for PFS after NACT-IDS and PDS. (DOCX 15 kb)

**Additional file 10: Table S8.** Multivariate analysis of risk factors for OS after NACT-IDS and PDS. (DOCX 16 kb)

## Data Availability

The datasets used and/or analysed during the current study are available from the corresponding author on reasonable request.
