# Supplementary material for: Evaluating the benefits of neoadjuvant chemotherapy for advanced epithelial ovarian cancer: a retrospective study
Source: J Ovarian Res. 2019 Sep 13;12:85. doi: 10.1186/s13048-019-0562-9 (PMC6744704; doi:10.1186/s13048-019-0562-9)
Supplement: Supplementary file 1 — Additional file 1: Table S1. Size comparison of the residual tumor between the PDS and NACT group. (DOCX 15 kb) [file 13048_2019_562_MOESM1_ESM.docx]

**Supplemental Table 1. Size comparison of the residual tumor between the PDS and NACT group**

| Size of residual disease | None (R0) | ＜1cm (R1) | ≥1cm(R2) |
| --- | --- | --- | --- |
| PDS group (n=141) | 32.6%(46/141) | 31.2%(44/141) | 36.2%(51/141) |
| NACT group (n=79) | 43.0% (34/79) | 35.4% (28/79) | 21.5% (17/79) |
| P value | 0.123 | 0.520 | 0.024 |
