# Supplementary material for: Evaluating the benefits of neoadjuvant chemotherapy for advanced epithelial ovarian cancer: a retrospective study
Source: J Ovarian Res. 2019 Sep 13;12:85. doi: 10.1186/s13048-019-0562-9 (PMC6744704; doi:10.1186/s13048-019-0562-9)
Supplement: Supplementary file 2 — Additional file 2: Table S2. Univariate analysis of risk factors for platinum resistance recurrence after NACT-IDS and PDS. (DOCX 18 kb) [file 13048_2019_562_MOESM2_ESM.docx]

Supplemental Table 2. Univariate analysis of risk factors for platinum resistance recurrence after NACT-IDS and PDS.

| Characteristics | Chemo-sensitive group  (n=146) | Chemo-resistant group (n=61) | *P* value |
| --- | --- | --- | --- |
| Age (years), mean±SD | 55.39±10.38 | 57.49±11.65 | 0.288 |
| Pathology type |  |  | 0.009 |
| Serous carcinoma | 74.1% (129/174) | 25.9% (45/174) |  |
| Other types | 51.5% (17/33) | 48.5% (16/33) |  |
| Stage |  |  | 0.975 |
| IIIC | 70.5% (117/166) | 29.5% (49/166) |  |
| IV | 70.7% (29/41) | 29.3% (12/41) |  |
| Histology grade* |  |  | 0.420 |
| G1 | 100.0% (2/2) | 0% (0/2) |  |
| G2 | 77.4% (24/31) | 22.6% (7/31) |  |
| G3 | 69.0% (118/171) | 31.0% (53/171) |  |
| Initial CA125 level* |  |  | 0.268 |
| ＜500U/ml | 75.0% (57/76) | 25.0% (19/76) |  |
| ≥500U/ml | 67.7% (88/130) | 32.3% (42/130) |  |
| Pelvic mass |  |  | 0.098 |
| ＜10cm | 68.2% (118/173) | 31.8% (55/173) |  |
| ≥10cm | 82.4% (28/34) | 17.6% (6/34) |  |
| Large volume Ascites |  |  | 0.011 |
| No | 77.9% (88/113) | 22.1% (25/113) |  |
| Yes | 61.7% (58/94) | 38.3% (36/94) |  |
| Pleural effusion |  |  | 0.282 |
| No | 71.8% (130/181) | 28.2% (51/181) |  |
| Yes | 61.5% (16/26) | 38.5% (10/26) |  |
| Liver metastasis |  |  | 0.924 |
| No | 70.4% (131/186) | 29.6% (55/186) |  |
| Yes | 71.4% (15/21) | 28.6% (6/21) |  |
| Tumor distribution |  |  | 0.001 |
| Localized | 65.5%(112/171) | 34.5%(59/171) |  |
| Diffuse | 94.4%(34/36) | 5.6%(2/36) |  |
| NACT |  |  | 0.009 |
| No | 76.9% (100/130) | 23.1% (30/130) |  |
| Yes | 59.7% (46/77) | 40.3% (31/77) |  |
| Lymphadenectomy |  |  | 0.079 |
| No | 60.4% (29/48) | 39.6% (19/48) |  |
| Yes | 73.6% (117/159) | 26.4% (42/159) |  |
| Macroscopic residual disease |  |  | 0.006 |
| No | 81.8%(63/77) | 18.2% (14/77) |  |
| Yes | 63.8%(83/130) | 36.2% (47/130) |  |

**Histology grade * refers to 3 cases with unknown histology grade due to difficult histology recognition. Initial CA125 level * refers to 1 case with unknown Initial CA125 level.**
