# Supplementary material for: Evaluating the benefits of neoadjuvant chemotherapy for advanced epithelial ovarian cancer: a retrospective study
Source: J Ovarian Res. 2019 Sep 13;12:85. doi: 10.1186/s13048-019-0562-9 (PMC6744704; doi:10.1186/s13048-019-0562-9)
Supplement: Supplementary file 3 — Additional file 3: Table S3. Multivariate analysis of risk factors for platinum resistance recurrence after NACT-IDS and PDS. (DOCX 16 kb) [file 13048_2019_562_MOESM3_ESM.docx]

**Supplemental Table 3. Multivariate analysis of risk factors for platinum resistance recurrence after NACT-IDS and PDS.**

| Factors | B value | OR value | 95%CI | P value |
| --- | --- | --- | --- | --- |
| NACT-IDS vs PDS | 0.883 | 2.417 | 1.218-4.796 | 0.012 |
| Serous vs nonserous histology | -0.985 | 0.373 | 0.166-0.842 | 0.018 |
| Macroscopic residual disease | 0.657 | 1.929 | 0.936-3.977 | 0.075 |
| **diffuse disseminated disease** | 0.199 | 1.220 | 0.535-2.781 | 0.637 |
| Large volume ascites | 0.558 | 1.748 | 0.905-3.374 | 0.096 |
