# Supplementary figures and images for: Evaluating the benefits of neoadjuvant chemotherapy for advanced epithelial ovarian cancer: a retrospective study
Source: J Ovarian Res. 2019 Sep 13;12:85. doi: 10.1186/s13048-019-0562-9 (PMC6744704; doi:10.1186/s13048-019-0562-9)

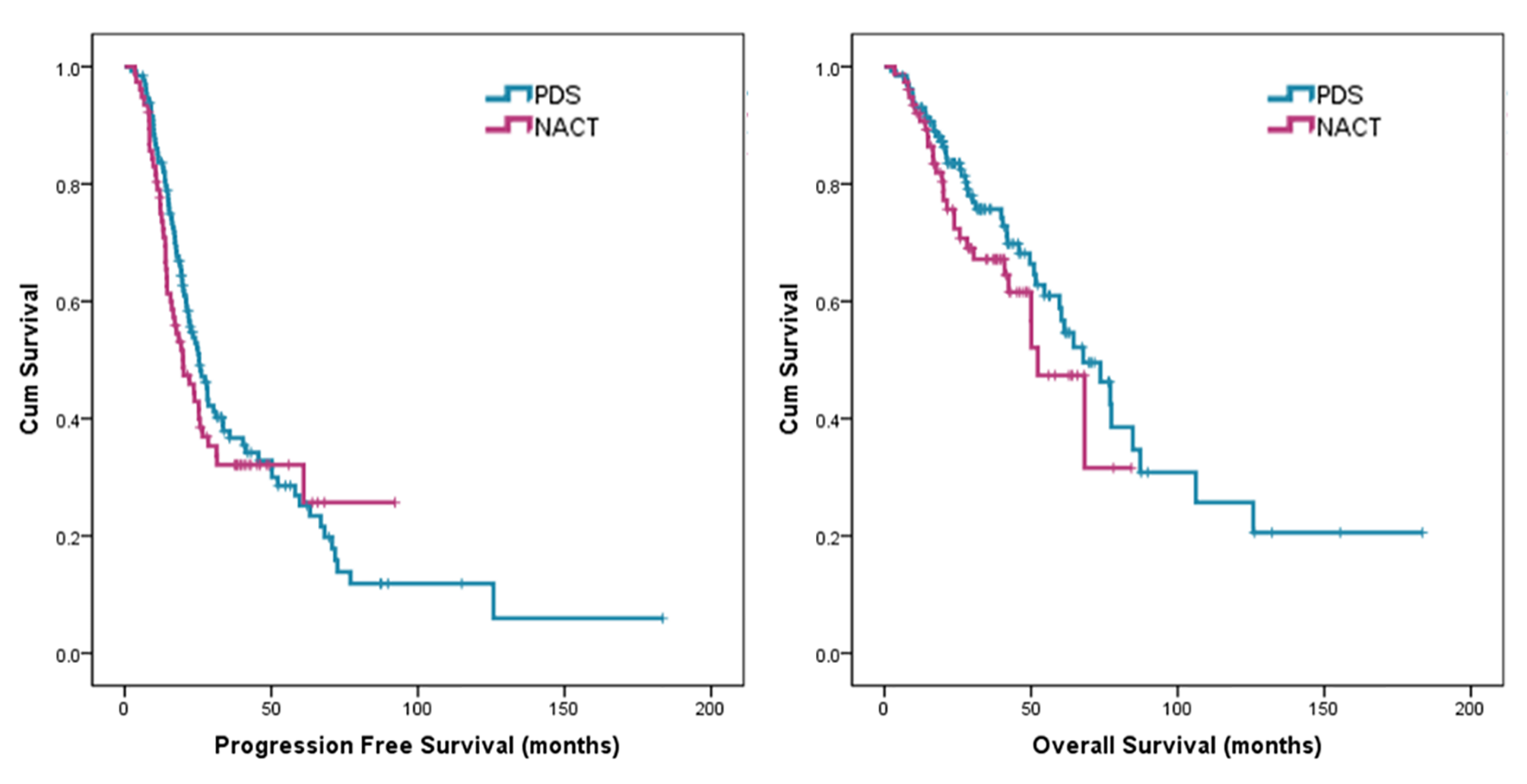

Supplement: Supplementary file 4 — Additional file 4: Figure S1. Kaplan–Meier survival curves for PFS(left) and OS(right) for the NACT and PDS groups. (TIF 285 kb) [file 13048_2019_562_MOESM4_ESM.tif]

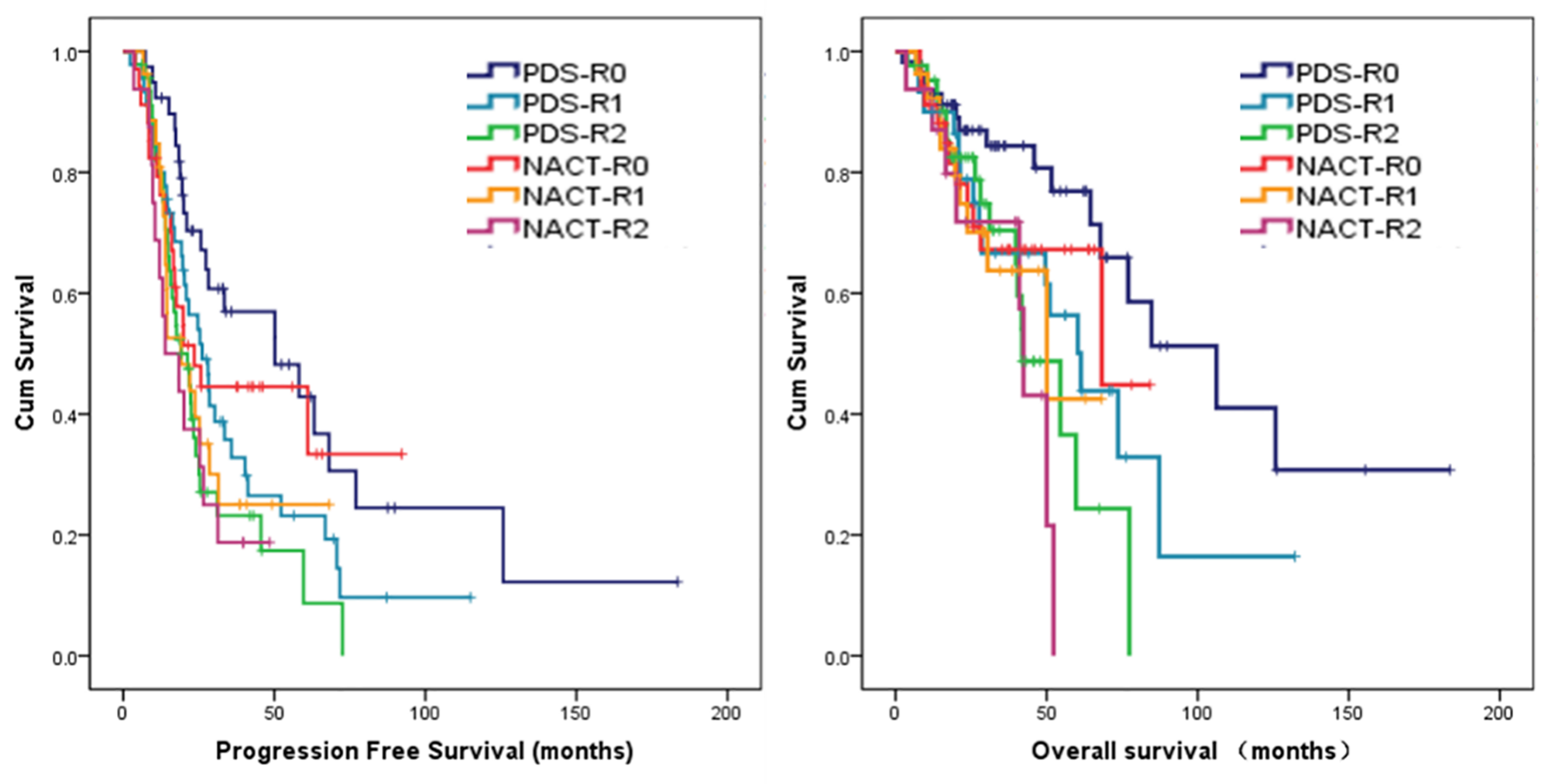

Supplement: Supplementary file 6 — Additional file 6: Figure S2. Kaplan–Meier survival curve for PFS(left) and OS(right) between the subgroups based on treatment selection and residual tumor. (TIF 478 kb) [file 13048_2019_562_MOESM6_ESM.tif]
