# Supplementary material for: Evaluating the benefits of neoadjuvant chemotherapy for advanced epithelial ovarian cancer: a retrospective study
Source: J Ovarian Res. 2019 Sep 13;12:85. doi: 10.1186/s13048-019-0562-9 (PMC6744704; doi:10.1186/s13048-019-0562-9)
Supplement: Supplementary file 5 — Additional file 5: Table S4. Prognosis comparison between the subgroups based on residual tumor for the PDS and NACT groups. (DOCX 16 kb) [file 13048_2019_562_MOESM5_ESM.docx]

Supplemental Table 4. Prognosis comparison between the subgroups based on residual tumor for the PDS and NACT groups

| Prognosis |  | Subgroups | |  | P value |
| --- | --- | --- | --- | --- | --- |
|  | PDS-R0 | PDS-R1 | | PDS-R2 |  |
| PFS | 50.2 (20.7-79.6) | 25.4 (17.2-33.6) | | 19.1 (13.0-25.3) | 0.001 |
| (months, 95%CI) | NACT-R0 | NACT-R1 | | NACT-R2 |  |
|  | 23.5 (13.0-34.1) | | 19.2 (7.5-31.0) | 13.8 (3.3-24.3) | 0.266 |
|  | PDS-R0 | | PDS-R1 | PDS-R2 |  |
| OS | 106.2 (54.9-157.5) | 61.3 (41.1-81.6) | | 41.8 (28.8-54.9) | 0.001 |
| (months, 95%CI) | NACT-R0 | NACT-R1 | | NACT-R2 |  |
|  | 68.2 (1.1-135.4) | 50.0 (16.2-83.9) | | 42.3 (39.0-45.6) | 0.269 |
