# Supplementary material for: Evaluating the benefits of neoadjuvant chemotherapy for advanced epithelial ovarian cancer: a retrospective study
Source: J Ovarian Res. 2019 Sep 13;12:85. doi: 10.1186/s13048-019-0562-9 (PMC6744704; doi:10.1186/s13048-019-0562-9)
Supplement: Supplementary file 7 — Additional file 7: Table S5. Univariate analysis of risk factors for PFS after NACT-IDS and PDS. (DOCX 18 kb) [file 13048_2019_562_MOESM7_ESM.docx]

Supplemental Table 5. Univariate analysis of risk factors for PFS after NACT-IDS and PDS.

| Characteristics | Median PFS | 95% CI | *P* value |
| --- | --- | --- | --- |
| Age (years) |  |  | 0.482 |
| ＜45  ≥45 | 23.54  24.03 | 11.41-35.67  20.07-27.99 |  |
| Pathology type |  |  | 0.430 |
| Serous carcinoma | 23.74 | 20.25-27.23 |  |
| Other types | 22.52 | 9.75-35.29 |  |
| Stage |  |  | 0.887 |
| IIIC | 24.99 | 20.99-28.99 |  |
| IV | 22.52 | 14.32-30.72 |  |
| Initial CA125 level* |  |  | 0.587 |
| ＜500U/ml | 24.03 | 19.67-28.39 |  |
| ≥500U/ml | 22.52 | 17.59-27.45 |  |
| Pelvic mass |  |  | 0.095 |
| ＜10cm | 46.19 | 33.05-59.33 |  |
| ≥10cm | 43.04 | 36.27-49.81 |  |
| Large volume Ascites |  |  | 0.553 |
| No | 43.1 | 37.41-48.79 |  |
| Yes | 48.46 | 30.05-66.87 |  |
| Pleural effusion |  |  | 0.215 |
| No | 24.66 | 21.77-27.55 |  |
| Yes  Tumor distribution  Localized  Diffuse | 15.62  28.18  21.70 | 8.83-22.41  21.76-34.60  17.63-25.77 | 0.197 |
| NACT |  |  | 0.371 |
| No | 25.38 | 21.12-29.64 |  |
| Yes | 19.89 | 13.41-26.37 |  |
| Chemoresistance |  |  | 0.000 |
| No | 33.53 | 17.28-49.78 |  |
| Yes | 10.75 | 9.42-12.08 |  |
| Macroscopic residual disease |  |  | 0.004 |
| No | 28.18 | 17.42-38.94 |  |
| Yes | 21.70 | 18.26-25.14 |  |

Initial CA125 level * refers to 1 case with unknown Initial CA125 level.
