# Supplementary material for: Evaluating the benefits of neoadjuvant chemotherapy for advanced epithelial ovarian cancer: a retrospective study
Source: J Ovarian Res. 2019 Sep 13;12:85. doi: 10.1186/s13048-019-0562-9 (PMC6744704; doi:10.1186/s13048-019-0562-9)
Supplement: Supplementary file 8 — Additional file 8: Table S6. Univariate analysis of risk factors for OS after NACT-IDS and PDS. (DOCX 19 kb) [file 13048_2019_562_MOESM8_ESM.docx]

Supplemental Table 6. Univariate analysis of risk factors for OS after NACT-IDS and PDS.

| Characteristics | Median PFS | 95% CI | *P* value |
| --- | --- | --- | --- |
| Age (years) |  |  | 0.895 |
| ＜45  ≥45 | 62.25  78.76 | 47.81-76.69  63.16-95.501 |  |
| Pathology type |  |  | 0.363 |
| Serous carcinoma | 80.24 | 63.62-96.86 |  |
| Other types | 64.58 | 47.74-81.42 |  |
| Stage |  |  | 0.435 |
| IIIC | 76.55 | 60.98-92.12 |  |
| IV | 88.14 | 65.31-110.96 |  |
| Initial CA125 level* |  |  | 0.474 |
| ＜500U/ml | 77.00 | 57.41-96.59 |  |
| ≥500U/ml | 60.30 | 47.05-73.55 |  |
| Pelvic mass |  |  | 0.294 |
| ＜10cm | 67.76 | 51.54-83.98 |  |
| ≥10cm | 64.50 | 30.95-89.06 |  |
| Large volume Ascites |  |  | 0.008 |
| No | 77.00 | 61.69-92.31 |  |
| Yes | 51.58 | 40.71-62.45 |  |
| Pleural effusion |  |  | 0.903 |
| No | 67.76 | 52.90-82.62 |  |
| Yes | 59.70 | 27.84-91.57 |  |
| Tumor distribution  Localized  Diffuse  NACT | 77.00  60.30 | 60.30-93.71  42.79-77.81 | 0.136  0.209 |
| No | 67.76 | 52.95-82.57 |  |
| Yes | 52.27 | 39.85-64.69 |  |
| Chemoresistance |  |  | 0.000 |
| No | 77.36 | 62.03-92.69 |  |
| Yes | 20.09 | 17.61-22.57 |  |
| Macroscopic residual disease |  |  | 0.003 |
| No | 84.69 | 50.19-110.20 |  |
| Yes | 50.04 | 40.34-59.74 |  |

Initial CA125 level * refers to 1 case with unknown Initial CA125 level.
