# Supplementary material for: Evaluating the benefits of neoadjuvant chemotherapy for advanced epithelial ovarian cancer: a retrospective study
Source: J Ovarian Res. 2019 Sep 13;12:85. doi: 10.1186/s13048-019-0562-9 (PMC6744704; doi:10.1186/s13048-019-0562-9)
Supplement: Supplementary file 9 — Additional file 9: Table S7. Multivariate analysis of risk factors for PFS after NACT-IDS and PDS. (DOCX 15 kb) [file 13048_2019_562_MOESM9_ESM.docx]

**Additional file 9: Table s7. Multivariate analysis of risk factors for PFS after NACT-IDS and PDS.**

| Factors | B value | OR value | 95%CI | P value |
| --- | --- | --- | --- | --- |
| NACT | 0.401 | 1.042 | 0.726-1.496 | 0.823 |
| Chemoresistance | 1.999 | 7.378 | 4.928-11.046 | 0.000 |
| Pleural Effusion | -0.176 | 0.839 | 0.506-1.390 | 0.495 |
| Large volume ascites | 0.276 | 1.318 | 0.927-1.874 | 0.124 |
| Pelvic Mass＞10cm | -0.056 | 0.946 | 0.576-1.553 | 0.826 |
| Macroscopic residual disease | 0.195 | 1.216 | 0.810-1.824 | 0.346 |
