# Supplementary material for: Evaluating the benefits of neoadjuvant chemotherapy for advanced epithelial ovarian cancer: a retrospective study
Source: J Ovarian Res. 2019 Sep 13;12:85. doi: 10.1186/s13048-019-0562-9 (PMC6744704; doi:10.1186/s13048-019-0562-9)
Supplement: Supplementary file 10 — Additional file 10: Table S8. Multivariate analysis of risk factors for OS after NACT-IDS and PDS. (DOCX 16 kb) [file 13048_2019_562_MOESM10_ESM.docx]

**Supplemental Table 8. Multivariate analysis of risk factors for OS after NACT-IDS and PDS.**

| Factors | B value | OR value | 95%CI | P value |
| --- | --- | --- | --- | --- |
| NACT | 0.203 | 1.225 | 0.741-2.023 | 0.429 |
| Chemoresistance | 1.961 | 7.106 | 4.197-12.031 | 0.000 |
| Macroscopic residual disease | 0.642 | 1.899 | 1.063-3.394 | 0.030 |
| Serous vs nonserous histology | 0.223 | 1.249 | 0.650-2.402 | 0.504 |
| Pleural Effusion | -0.351 | 0.704 | 0.344-1.440 | 0.336 |
| Large volume ascites | 0.035 | 1.036 | 0.628-1.709 | 0.891 |
